# Patient‐derived iPS cells to study TNT‐mediated interaction between microglia and neurons

**DOI:** 10.1002/alz70855_102361

**Published:** 2025-12-23

**Authors:** Dimitri Budinger

**Affiliations:** ^1^ University of Luxembourg, Luxembourg, Luxembourg

## Abstract

**Background:**

Microglia are crucial for maintaining brain health and neuronal function. A novel mechanism of microglia‐neuron interaction, mediated by direct intercellular connections, namely through tunnelling nanotubes (TNTs), has recently been described between these cell types (Scheiblich et al., 2024). Preliminary data in mice cells suggest that microglia can form TNTs with neurons and free those from pathological protein aggregates made of hyperphosphorylated tau or α‐syn, and promoting protein degradation (Scheiblich et al., 2024). Additionally, microglia were shown to share their healthy mitochondria with burdened neurons through TNT, reducing oxidative stress and restoring neuronal health. Failure of such a neuroprotective mechanism may, in turn, be causative for neurodegeneration. Beyond supporting individual cells, TNT‐mediated interactions enhance the collective function of microglial and neuronal populations, mitigating inflammation and neuronal dysfunction.

**Method:**

We employ induced pluripotent stem cells (iPSCs) derived from Alzheimer's disease patients and healthy controls. These iPSCs are differentiated into cortical neurons and microglia, then cocultured to investigate TNT formation and the dynamics of aggregated protein transfer between these cell types, using high resolution microscopy techniques. Additionally, we evaluate how disease genotypes influence TNT formation parameters.

**Result:**

Our findings confirm the presence of TNTs between iPSC‐derived neurons and microglia. We show that the genetic background of these cells influences TNT morphology, including their length, diameter, and the number of TNTs per cell. Live imaging analyses further reveal that TNT dynamics—such as their formation speed, persistence over time, and the transfer kinetics of aggregated proteins and organelles—differ between control and patient‐derived cultures.

**Conclusion:**

In conclusion, this study confirms the formation of tunneling nanotubes (TNTs) between iPSC‐derived neurons and microglia, revealing their role in clearing pathological protein aggregates and exchanging organelles. Genetic background significantly influences TNT morphology and dynamics, affecting neuroprotective processes. These findings deepen our understanding of microglia‐neuron interactions and highlight TNTs as potential therapeutic targets in neurodegenerative diseases.

